# Association of Preoperative Midazolam Administration With Patient Anxiety During the Transition From the Preoperative Holding Bay to the Operating Room: A Prospective Observational Study

**DOI:** 10.7759/cureus.115462

**Published:** 2026-08-30

**Authors:** Tina Ramishvili, Rafi Khandaker, Jan N Schwarz, Samira Masoumi, Constance Rippel, Dorothee Stratz, Osamah M Zmily, Lorenz Droege, Ibraheem M Karaye, Nafiu O Olubukola, Aiman Suleiman, Jonathan E Alpert, Simon A Rego, Robert Ruemmler, Karuna Wongtangman, Matthias Eikermann, Philipp Fassbender

**Affiliations:** 1 Department of Anesthesiology, Albert Einstein College of Medicine, Montefiore Medical Center, Bronx, USA; 2 Department of Anesthesiology and Reanimation, Essen University Hospital, Essen, DEU; 3 Department of Anesthesiology, University Children's Hospital Zurich, Zurich, CHE; 4 Department of Psychiatry and Behavioral Sciences, Albert Einstein College of Medicine, Montefiore Medical Center, Bronx, USA

**Keywords:** anxiolysis, midazolam, perioperative care, postoperative recall, preoperative anxiety

## Abstract

Background

Among patients awaiting surgical procedures, up to 80% experience anxiety. Midazolam is commonly administered in the preoperative holding bay to reduce anxiety. However, the effect of benzodiazepines can last longer than surgery and may lead to an increased risk of neurocognitive impairment. Thus, we looked into the effects of midazolam administration beyond the preoperative holding bay. We hypothesized that omitting midazolam in the holding bay increases anxiety in the operating room and the risk of unpleasant explicit recall.

Methods

This study included adult patients undergoing surgical procedures at Montefiore Medical Center (June-August 2024). Anxiety was assessed using the anxiety component of the Amsterdam Preoperative Anxiety and Information Scale, and midazolam was administered accordingly. We assessed postoperative unpleasant explicit recall in a follow-up survey. Mixed-effects linear and modified Poisson regression models were used for analyses.

Results

Among 765 patients studied, 27.5% received midazolam. The mean (standard deviation (SD)) anxiety level was 7.8 (4.3) in the holding bay and 7.9 (4.7) in the operating room. Compared to the non-midazolam group, those receiving midazolam experienced decreased anxiety from the preoperative holding bay to the operating room (adjusted mean difference (AMD): -2.0; 95% confidence interval (CI): -2.5 to -1.5; p<0.001). The association was stronger in female patients (AMD: -2.9; 95% CI: -3.5 to -2.3; p<0.001) versus male patients (AMD: -0.9; 95% CI: -1.6 to -0.1; p=0.029) and in younger (≤30) patients (AMD: -3.8; 95% CI: -5.1 to -2.6; p<0.001) versus older patients (AMD: -1.8; 95% CI: -2.4 to -1.3; p<0.001). In the follow-up cohort, midazolam demonstrated a trend towards reduced postoperative unpleasant explicit recall (adjusted risk ratio: 0.6; 95% CI: 0.4 to 1.1; p=0.103). The Richmond Agitation-Sedation Scale in the midazolam group (0 (0-0); median (interquartile range (IQR)) indicated effective anxiolysis without sedation.

Conclusions

In summary, our data show that anxiety levels increase from the preoperative holding bay to the operating room, an effect that was mitigated in patients who received a mean (SD) of 1.9 (0.6) mg of midazolam. This anxiolytic benefit was magnified in female and younger patients. These findings underscore the importance of continuous, structured anxiety assessments.

## Introduction

Among patients awaiting surgical procedures, up to 80% experience anxiety [1-6]. High anxiety levels have been linked to increased morbidity and mortality [7-9]. When a composite outcome of perioperative complications was analyzed, high preoperative anxiety was linked to an increased risk of perioperative complications in patients undergoing complex, cancer-related general surgeries [10,11].

Despite its prevalence and clinical consequences, preoperative anxiety is often under-assessed and undertreated [2]. Furthermore, there is limited research on the changes in levels of anxiety as patients transition through different stages of the perioperative process and how this change can be influenced. The preoperative holding bay, often the first point of contact on the day of surgery, is where patients undergo initial assessments and preparations [12,13]. However, the subsequent transition to the operating room and the urgency of the actual procedure may worsen anxiety levels, potentially affecting patients' psychological state.

Midazolam is commonly administered in the preoperative holding bay to reduce anxiety [14]. However, this strategy may fail to identify patients who appear calm in the preoperative holding bay, but whose anxiety increases upon transition to the operating room, potentially affecting postoperative psychological outcomes. In this study, we measured anxiety and sedation levels during the transition from the preoperative holding bay to the operating room [15]. We hypothesized that patients who did not receive midazolam in the preoperative holding bay would have a significant increase in anxiety levels during this transition, challenging the conventional practice of administering midazolam solely based on anxiety observed in the preoperative holding bay [15]. In a follow-up survey, we further tested the hypothesis that not administering midazolam in the preoperative holding bay was associated with unpleasant explicit recall. This approach connects preoperative anxiolysis with long-term psychological outcomes.

## Materials and methods

Study design and population

We conducted a prospective observational, non-randomized, non-placebo study aimed at evaluating perioperative anxiety and its association with midazolam administration at Montefiore Medical Center between June 17, 2024, and August 22, 2024. The initial prospective observational study protocol was approved by the Albert Einstein College of Medicine Institutional Review Board at Montefiore Medical Center before the start of any study procedures under the local IRB# 2024-16360. The follow-up survey on unpleasant explicit recall after surgery was conducted after recruitment of the primary study was concluded. The follow-up survey study protocol was approved by the Albert Einstein College of Medicine Institutional Review Board at Montefiore Medical Center before the start of any study procedures under the local IRB# 2024-16551. For both studies, the Albert Einstein College of Medicine Institutional Review Board at Montefiore Medical Center granted a waiver for written informed consent and required only verbal consent to be obtained prior to study enrollment and participation. All methods were carried out in accordance with relevant guidelines and regulations. The study design and reporting adhere to the Strengthening the Reporting of Observational Studies in Epidemiology (STROBE) guidelines [16]. Patients or the public were not involved in the design, conduct, reporting, or dissemination plans of our research.

Adult patients presenting to the preoperative holding bay during the study period were consecutively approached for participation. Following verbal consent, the attending anesthesiologist performed the routine preoperative evaluation, including assessment of the patient's anxiety using the Amsterdam Preoperative Anxiety and Information Scale (APAIS) [17], which consists of four questions about anxiety related to surgery and anesthesia, rated on a 5-point scale (1="not at all worried" to 5="extremely worried"). This tool quantifies perioperative anxiety, with total anxiety scores ranging from 4 to 20 points [17]. Based on this assessment and routine clinical judgment, the attending anesthesiologist determined whether preoperative midazolam should be administered (Figure 1). For patients who received midazolam in the preoperative holding bay, the sedative effects of midazolam in the preoperative holding bay were evaluated using the Richmond Agitation-Sedation Scale (RASS) [18,19]. Upon arrival in the operating room and prior to induction of anesthesia, anxiety was reassessed using the APAIS [17]. At our institution, the standard transfer time from the preoperative holding bay to the operating room following midazolam administration is 20-30 minutes, though normal perioperative workflow variations can extend this duration.

**Figure 1:**
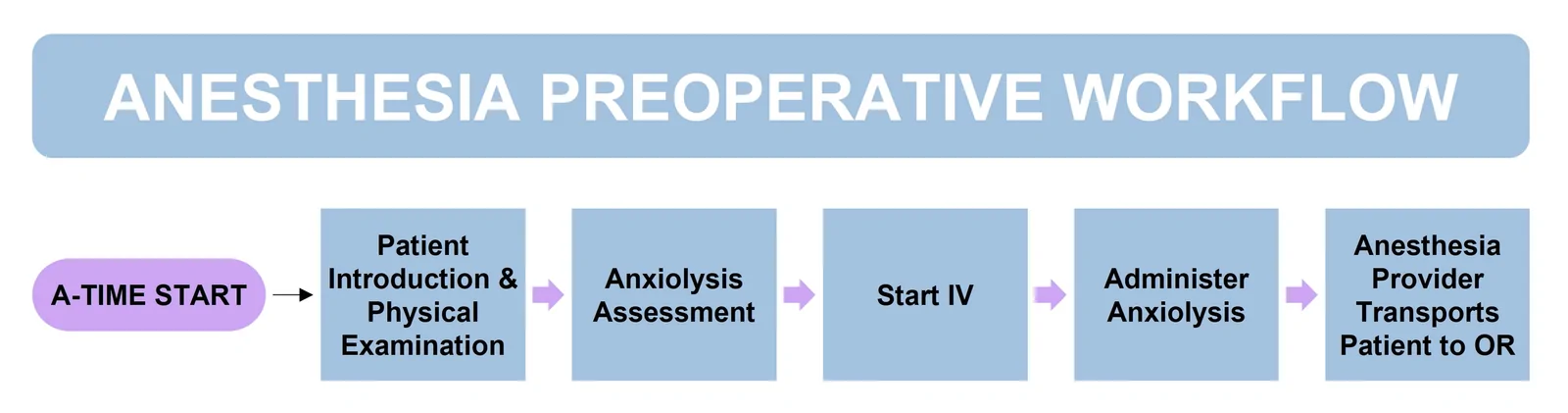
Structured, preoperative workflow at Montefiore Health Care Network Following the completion of the pre-anesthesia assessment, anesthesia time begins when the anesthesia team assumes continuous care of the patient and initiates preparation for anesthesia. The standardized workflow includes assessment of preoperative anxiety, placement of intravenous access, administration of an anxiolytic medication when indicated, and continued monitoring of the patient and the response to anxiolysis. The anesthesia provider subsequently accompanies and transports the patient to the OR, maintaining continuity of care throughout the preoperative period. OR: operating room

Three research assistants supported the study procedures by facilitating data collection, documenting the APAIS and RASS scores assigned during routine clinical care, and recording the subsequent operating room anxiety assessment. Before study initiation, all research assistants underwent standardized training in study procedures, data collection, and the use of the APAIS and RASS to ensure consistent documentation across study personnel.

Midazolam was administered intravenously (IV) as a single bolus (typically 0.02-0.03 mg kg^-1^) to achieve effective anxiolysis. Consequently, research assistants were aware of whether midazolam had been administered when conducting the subsequent operating room assessment, and outcome assessment was therefore not blinded. Patients were excluded only if we were unable to obtain at least two anxiety measurements at the predefined measurement points in the preoperative holding bay and the operating room. No imputation of missing data was performed.

Patient demographics, including age, sex, weight, height, type of surgical procedure, anesthesia type, and necessary variables for the confounder model, were extracted from electronic medical records to complement our observational data.

We contacted patients 9-11 months after their surgery to assess their explicit recall and asked them to report the first observation upon arrival to both the operating room and the post-anesthesia care unit (PACU) in a follow-up survey (Appendix A). From these responses, we created a composite outcome of unpleasant explicit recall, defined as the presence of any of the following either in the operating room or in the PACU: recall of bright lights, feeling cold, shivering, anxiety, panicking feeling, or pain. These types of explicit recall have been previously described in the literature [20,21].

Covariates and confounders

Potential confounders were identified based on clinical judgment and existing literature. In our statistical analysis, we controlled for the following a priori-defined variables that could potentially influence preoperative anxiety: age, sex, race/ethnicity, pre-existing comorbidities, American Society of Anesthesiologists (ASA) status, surgical service type, surgery classification, duration of surgery, type of anesthesia, admission status, and Montefiore Health System campus where the procedure was performed. If there was no linear relationship with the primary outcome, the covariates were grouped into quintiles or clinically significant categories prior to being included in the confounder model (Appendix B and Appendix C).

Statistical analysis

The primary outcome was the change in anxiety levels from the preoperative holding bay to the operating room. To account for the repeated measurements obtained from each participant and the hierarchical structure of the data, we used a multilevel mixed-effects linear regression model. This approach accounts for the correlation of repeated observations within individuals while allowing for clustering across patient ID, surgical services, and Montefiore Health System campuses. A post-hoc subgroup analysis was conducted in patients exhibiting high baseline anxiety defined as initial APAIS ≥11 in the holding bay [17]. Interaction terms between midazolam administration and sex, as well as age (≤30 versus >30 years) [22,23], were prespecified to evaluate potential effect modification.

The association between midazolam administration and unpleasant postoperative recall was evaluated using a modified Poisson regression model with robust error variances. Because the outcome was binary and not rare, this approach was selected to directly estimate adjusted relative risks, which are more clinically interpretable than odds ratios derived from logistic regression. No formal a priori power analysis was performed for the exploratory follow-up survey. Based on a post-hoc assessment, detecting a 6% difference between groups with 80% power (α=0.05) would require a total sample of approximately 1,360 patients; therefore, the current follow-up cohort (n=267) is presented as an exploratory analysis of potential trends.

Categorical variables were presented as frequency and column percentage. Continuous variables were summarized as mean (standard deviation) (SD) or median and interquartile range (IQR). All statistical analyses were performed using Stata Version 18 (StataCorp LLC, College Station, Texas, United States) and GraphPad Prism Version 10.0 (GraphPad Software, Boston, Massachusetts, United States).

## Results

Baseline characteristics

Seven hundred and sixty-five patients completed the anxiety assessments and were included in the final analysis (Figure 2). Demographics, comorbidities, and surgical treatment details are provided in Table 1. Among these 765 patients, 210 (27.5%) received a single IV bolus of midazolam in the preoperative holding bay, with a mean (SD) dose of 1.9 (0.6) mg. The time between midazolam administration and anxiety assessment in the operating room was 23.5 (30.1) minutes.

**Figure 2:**
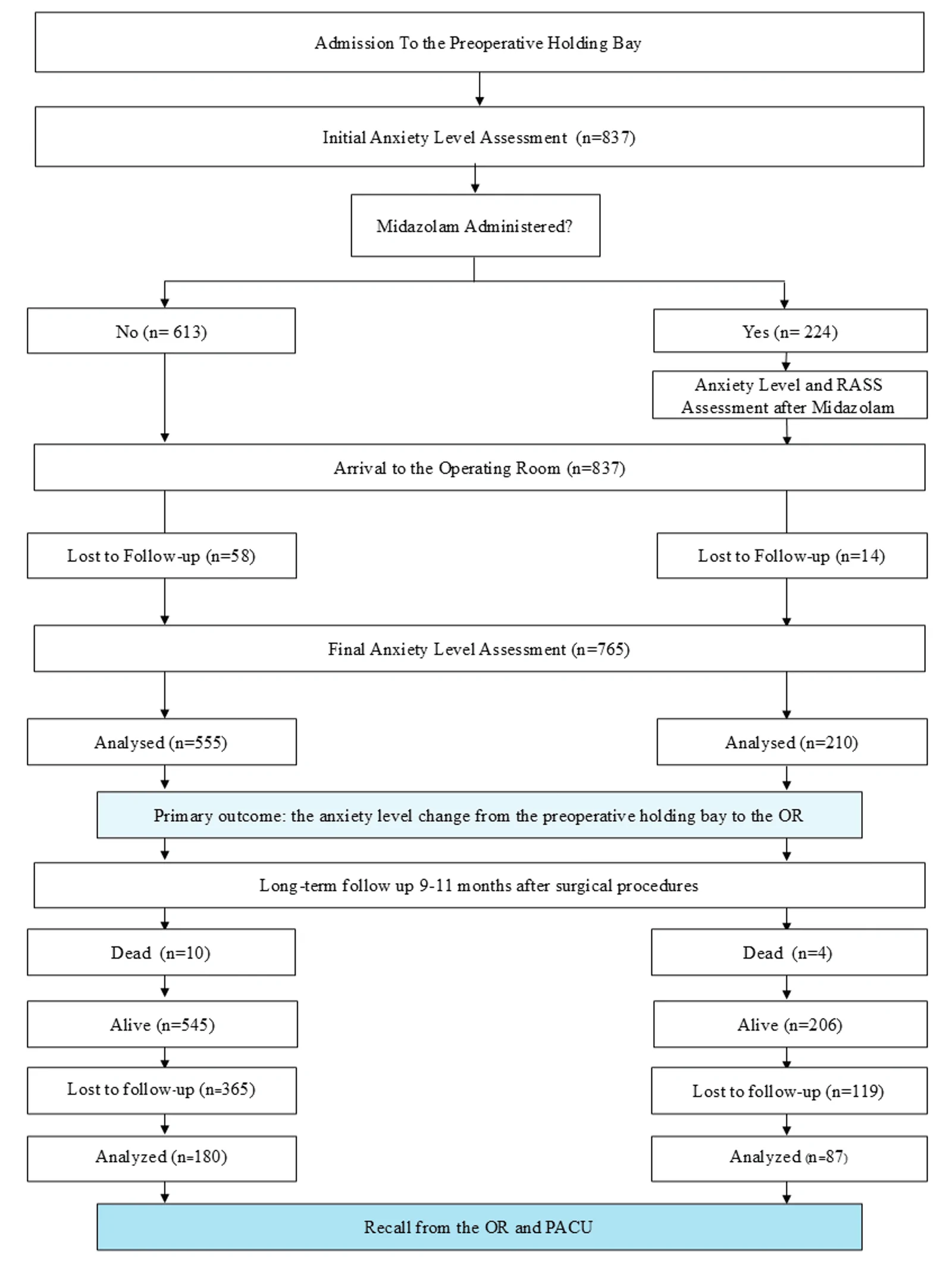
Study flow RASS: Richmond Agitation-Sedation Scale; OR: operating room; PACU: post-anesthesia care unit

**Table 1:** Baseline characteristics of the study population grouped by midazolam administration Data are presented as mean (SD) or median (IQR) for continuous measures and n (%) for categorical measures. ASA: American Society of Anesthesiologists; BMI: body mass index; ENT: ear, nose, and throat; HIV: human immunodeficiency virus

Characteristics	No midazolam	Midazolam
	N=555	N=210
Baseline demographics		
Female	353 (63.6%)	121 (57.6%)
Male	202 (36.4%)	89 (42.4%)
Age	53.4 (16.0)	49.3 (15.5)
BMI (kg m^-2^)	29.6 (25.2-37.1)	30.4 (25.7-34.8)
Weight (kg)	83.5 (68.8-101.0)	83.7 (69.0-100.0)
Race/ethnicity		
White	71 (12.8%)	24 (11.4%)
Hispanic	246 (44.3%)	92 (43.8%)
Black	136 (24.5%)	55 (26.2%)
Asian	16 (2.9%)	5 (2.4%)
Other	38 (6.8%)	11 (5.2%)
Not reported/unavailable	48 (8.6%)	23 (11%)
Surgical service		
Cardiac	25 (4.5%)	2 (1%)
ENT	54 (9.7%)	12 (5.7%)
Gastroenterology	18 (3.2%)	1 (0.5%)
General surgery	170 (30.6%)	49 (23.3%)
Gynecology	25 (4.5%)	5 (2.4%)
Neurosurgery	41 (7.4%)	5 (2.4%)
Orthopedics	69 (12.4%)	80 (38.1%)
Plastics	24 (4.3%)	11 (5.2%)
Thoracic	2 (0.4%)	1 (0.5%)
Transplant	5 (0.9%)	11 (5.2%)
Urology	38 (6.8%)	8 (3.8%)
Vascular	59 (10.6%)	19 (9%)
Other	25 (4.5%)	6 (2.9%)
Admission status		
Ambulatory	260 (46.8%)	144 (68.6%)
Same-day admission	259 (46.7%)	58 (27.6%)
Inpatient	36 (6.5%)	8 (3.8%)
Type of surgery		
Elective	532 (95.9%)	203 (96.7%)
Emergent	3 (0.5%)	1 (0.5%)
Urgent	20 (3.6%)	6 (2.9%)
Regional anesthesia	7 (1.2%)	68 (32.4%)
ASA status	3.0 (2.0-3.0)	2.0 (2.0-3.0)
Duration of surgery (min)	123.5 (114.7)	133.4 (106.8)
Midazolam dose (mg)	0	1.9 (0.6)
Charlson Comorbidity Index	1.0 (0.0-2.0)	0.0 (0.0-1.0)
Cancer	67 (12.1%)	23 (11%)
Congestive heart failure	68 (12.3%)	12 (5.7%)
Chronic pulmonary disease	120 (21.6%)	44 (21%)
Chronic vascular disease	39 (7%)	8 (3.8%)
Dementia	5 (0.9%)	2 (1%)
Diabetes mellitus with organ failure	82 (14.8%)	23 (11%)
Hemiparesis	9 (1.6%)	0 (0%)
Diabetes mellitus without organ failure	122 (22%)	45 (21.4%)
HIV	13 (2.3%)	4 (1.9%)
Myocardial infarction	32 (5.8%)	9 (4.3%)
Mild liver disease	40 (7.2%)	16 (7.6%)
Peripheral vascular disease	106 (19.1%)	24 (11.4%)
Renal failure	72 (13%)	22 (10.5%)
Rheumatoid arthritis	7 (1.3%)	5 (2.4%)

Anxiety measurements

The mean (SD) anxiety level in the overall cohort was 7.8 (4.3) in the preoperative holding bay and 7.9 (4.7) in the operating room. Values for each subcomponent of the APAIS are presented in Appendix D.

Compared to the non-midazolam group, patients who received midazolam experienced a decrease in anxiety levels (adjusted mean difference (AMD): -2.0; 95% CI: -2.5 to -1.5; p<0.001) from the preoperative holding bay to the operating room.

Among patients who did not receive midazolam, the mean (SD) anxiety levels were 7.3 (4.0) in the preoperative holding bay and 8.0 (4.6) in the operating room, with an AMD of 0.6 (95% CI: 0.4 to 0.9; p<0.001) (Figure 3; Appendix E). Midazolam decreased patients' anxiety level from the preoperative holding bay to the operating room from 9.1 (5.0) to 7.7 (4.9) with an AMD of -1.4 (95% CI: -1.9 to -0.9; p<0.001) (Figure 3; Appendix E). The mixed-effects restricted maximum likelihood (REML) model included 1,530 observations from 765 patients. The model fit was significant (Wald χ² (53)=179.45; p<0.001), with a log restricted likelihood of -4010.25 (Akaike Information Criterion (AIC): 8136.50; Bayesian Information Criterion (BIC): 8445.81). The mixed model provided a significantly better fit than a standard linear model (LR χ² (3)=696.23; p<0.001). Patient-level random intercept variance was 4.88, and residual variance was 4.20.

**Figure 3:**
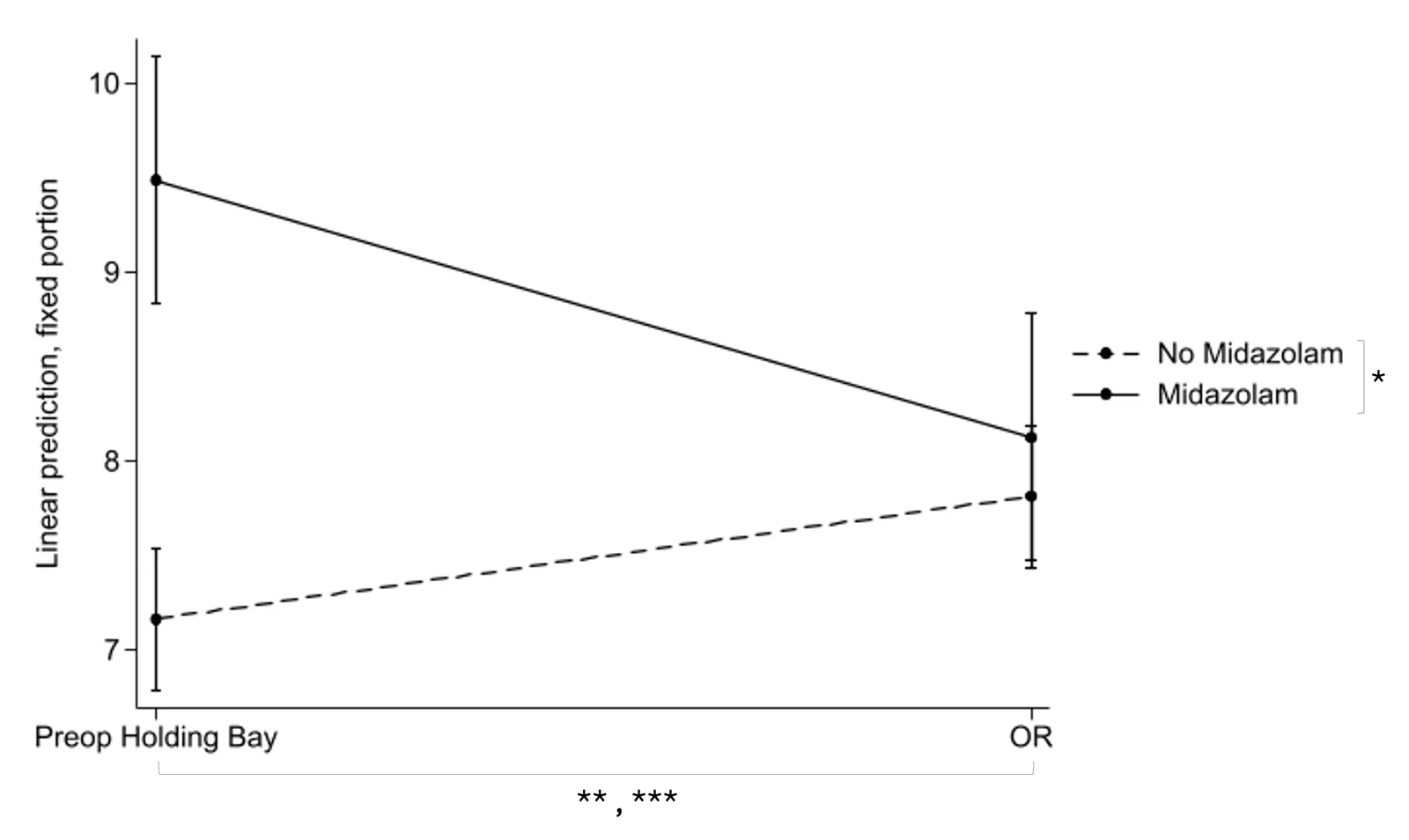
Change in anxiety level from the preoperative holding bay to the OR Adjusted anxiety levels from the preoperative holding bay to the OR for patients who received midazolam (solid black line) and those who did not (dashed black line). Values on the y-axis represent adjusted predicted anxiety scores based on the fixed effects of a multilevel linear model. Error bars represent 95% CI. Compared to the non-midazolam group, patients who received midazolam experienced a decrease in anxiety levels (AMD: -2.0; 95% CI: -2.5 to -1.5; p<0.001*) from the holding bay to the OR. Patients who did not receive midazolam experienced an increase in anxiety level from the preoperative holding bay to the OR with an AMD of 0.6 (95% CI: 0.4 to 0.9; p<0.001**). The between-group difference in change in anxiety was -1.4 (95% CI: -1.9 to -0.9; p<0.001***). * indicates the significant difference in the anxiety score between patients who received and did not receive midazolam (p<0.001). ** indicates the significant increase in anxiety levels within the non-midazolam group (from the holding bay to the OR) (p<0.001). *** indicates the significant decrease in anxiety levels within the midazolam group (from the holding bay to the OR) (p<0.001). OR: operating room; AMD: adjusted mean difference

Furthermore, in a post-hoc subgroup analysis restricted to patients exhibiting high baseline anxiety [15] (initial APAIS ≥11 in the holding bay (n=193), of whom 71 received midazolam), midazolam administration was associated with a significantly greater reduction in anxiety upon transition to the operating room compared to those who did not receive midazolam (AMD: -3.7; 95% CI: -4.8 to -2.5; p<0.001).

Female patients exhibited higher anxiety levels compared to their male counterparts both in the preoperative holding bay (8.4 (4.6) versus 6.9 (3.8); p<0.001) and in the operating room (8.6 (4.9) versus 7.0 (4.1); p<0.001). The association of midazolam with anxiety was modified by sex and age (p for interaction <0.001 and 0.003, respectively). Among female patients, the AMD was -2.9 (95% CI: -3.5 to -2.3; p<0.001) compared to -0.9 (95% CI: -1.6 to -0.1; p=0.029) in male patients. Similarly, the association was stronger among younger patients (≤30 years), with an AMD of -3.8 (95% CI: -5.1 to -2.6; p<0.001) compared with -1.8 (95% CI: -2.4 to -1.3; p<0.001) among those older than 30 years (Table 2; Figure 4).

**Table 2:** Association of midazolam administration with anxiety levels and interaction with age and sex OR: operating room; AMD: adjusted mean difference

		Anxiety in the preoperative holding bay baseline (mean±SD)	Anxiety in the OR (mean±SD)	AMD/95% CI/p-value	P-value for interaction
Effect modification of midazolam on the association of sex and anxiety level					
Male (N=291)					<0.001
Midazolam not administered	n=202	6.3±3.3	6.7±3.8	-0.9/-1.6 to -0.1/0.029	
Midazolam administered	n=89	8.1±4.4	7.6±4.7		
Female (N=474)					
Midazolam not administered	n=353	7.9±4.2	8.7±4.8	-2.9/-3.5 to -2.3/<0.001	
Midazolam administered	n=121	9.8±5.3	7.8±5.0		
Effect modification of midazolam on the association of age and anxiety level					
≤30 years old (N=91)					0.003
Midazolam not administered	n=54	7.4±3.5	8.6±4.6	-3.8/-5.1 to -2.6/<0.001	
Midazolam administered	n=37	9.8±5.0	7.3±3.9		
>30 years old (N=674)					
Midazolam not administered	n=501	7.3±4.0	7.9±4.6	-1.8/-2.4 to -1.3/<0.001	
Midazolam administered	n=173	8.9±5.0	7.8±5.0		

**Figure 4:**
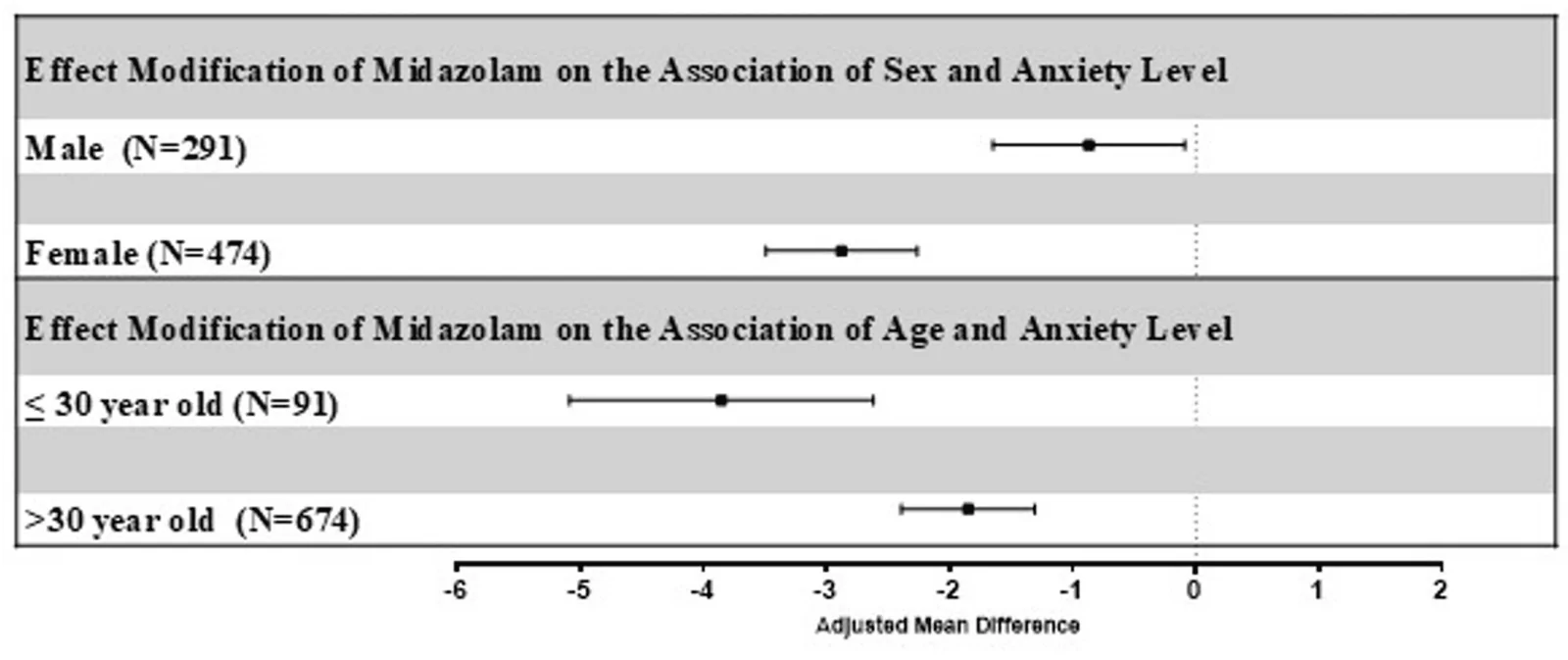
Forest plot illustrating the association of midazolam administration with anxiety levels and interaction with age and sex The forest plot illustrates the effect modifications between midazolam administration and sex and age. Sex (p-value for interaction: <0.001) and age (p-value for interaction: 0.003) modified the association between midazolam administration and anxiety level. The association of midazolam administration and decrease in anxiety level was greater in female (AMD: -2.9 (-3.5 to -2.3)) and in patients aged ≤30 (AMD: -3.8 (-5.1 to -2.6)). AMD: adjusted mean difference

Sedation measurements

The RASS was recorded in 95 (45%) patients who received midazolam, and the median (IQR) RASS was 0 (0-0), indicating no sedation (Figure 5). There were no differences in RASS scores between female and male patients (0 (0-0) versus 0 (0-0); p=0.868) or between younger and older patients (0 (0-0) versus 0 (0-0); p=0.431).

**Figure 5:**
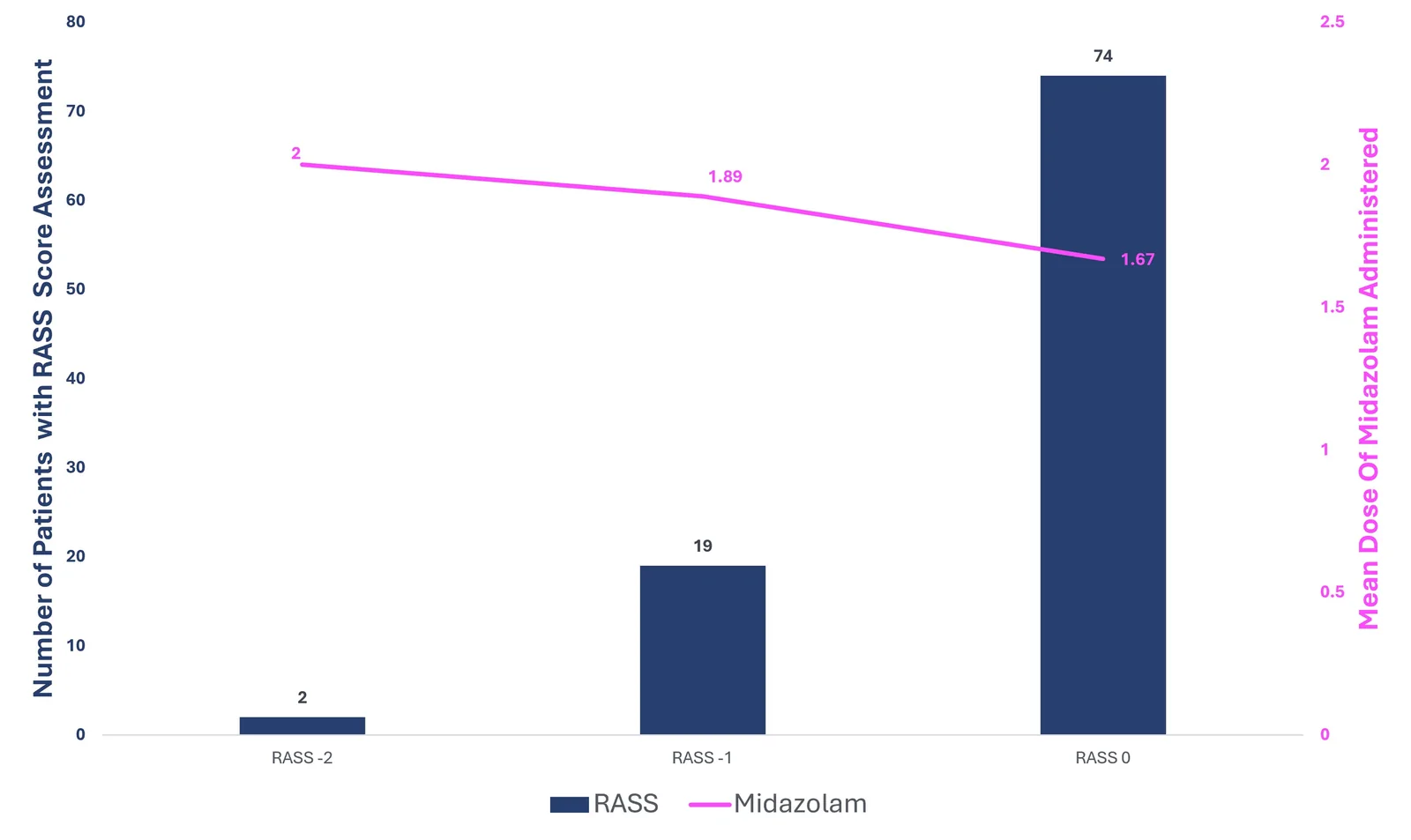
Distribution of RASS scores and associated midazolam dosing The bar graph (navy, left y-axis) shows the distribution of RASS scores among patients who received midazolam alone (n=95). The majority of patients (n=74) maintained a RASS score of 0 (alert and calm), while 19 patients had a RASS score of -1 (drowsy), and two patients had a RASS score of -2 (light sedation). The line graph (pink, right y-axis) depicts the mean midazolam dose (mg) administered to patients in each RASS category. RASS: Richmond Agitation-Sedation Scale

Postoperative recall

As part of the exploratory follow-up survey, 267 patients (34.9%) completed the postoperative recall questionnaires (Figure 2). Within this cohort, 180 patients had not received preoperative midazolam, while 87 had. Overall, 78 patients reported experiencing at least one unpleasant explicit recall in the operating room or PACU. Unpleasant recall was reported by 56 patients (31.1%) in the non-midazolam group, compared to 22 patients (25.3%) in the midazolam group. Midazolam administration demonstrated a non-significant trend towards reduced unpleasant explicit recall (adjusted risk ratio: 0.6; 95% CI: 0.4 to 1.1; p=0.103).

## Discussion

Our data show that anxiety levels increase from the preoperative holding bay to the operating room, an effect that was mitigated in patients who received a mean (SD) of 1.9 (0.6) mg of midazolam.

Consistent with the findings of Kumar and colleagues [24], our study demonstrated a significant increase in patients' anxiety during the transition from the preoperative holding bay to the operating room. Our study adds the information that this increase in anxiety can be prevented using anxiolytic therapy. Midazolam decreased preoperative anxiety levels and attenuated the increase in anxiety from the preoperative holding bay to the operating room. Similarly, when benzodiazepines were administered the night before and on the day of surgery, anxiety levels in the operating room were lower compared to those in the preoperative holding bay [25].

Midazolam is a well-established anxiolytic agent in perioperative contexts [3,14]. However, some clinicians are concerned about oversedation in an unmonitored setting in the preoperative holding bay [26]. Our institutional standard dosing for midazolam is comparatively lower than traditional premedication doses, a deliberate strategy, to maximize patient safety and mitigate risks of respiratory depression [14]. Consistent with this approach, midazolam did not induce sedation as measured by the RASS. Thus, in our institution, we do not request cardiorespiratory monitoring after midazolam administration when RASS remains -1 or higher. This approach is comparable to anxiolysis provided by clinicians in outpatient settings [27].

In our study, we observed a substantial variance in the time interval between midazolam administration and the subsequent anxiety assessment in the operating room. Although IV midazolam typically reaches peak clinical effect within five minutes and maintains a duration of action up to 80 minutes [28], the combination of this high variance and our comparatively lower midazolam doses likely contributed to the modest effect size observed in our cohort. 

The mean administered midazolam dose in our study was about 2 mg. Comparable doses have been shown to provide effective preoperative anxiolysis without causing adverse outcomes, such as delirium, even among older patient populations [29-31]. By contrast, higher doses (0.06 mg kg^-1^) have been associated with increased risk of complications, including oversedation and respiratory depression [32]. Furthermore, high-dose sedation in the postoperative and intensive care periods is a risk factor for delirium, mortality, and a loss of functional independence [33,34].

We found that female patients exhibited higher anxiety levels compared to males, despite there being no significant difference in weight-adjusted midazolam dosing between sexes, a phenomenon consistently reported in prior research, including the perioperative setting [17,25,35-39]. This has often been attributed to physiological factors such as fluctuations in estrogen and progesterone levels, which increase susceptibility to higher levels of anxiety [24,39]. Additionally, females are more likely to report anxiety than their male counterparts, and the anxiolytic effects of midazolam are magnified in female patients compared to males [25,32]. Midazolam's anxiolytic effects were magnified in younger patients aged 30 or younger, which is consistent with previous reports [32]. Younger populations may benefit from targeted pharmacological anxiolysis during the transition to the operating room, since the risk of respiratory side effects of midazolam is lower in younger patients without comorbidity burden [32].

In our follow-up survey, patients who received midazolam preoperatively were less likely to report symptoms associated with unpleasant explicit recall (such as recall of bright lights, feeling cold, shivering, anxiety, panicking feeling, or pain) compared with those who did not receive midazolam. Although the difference was not statistically significant, the adjusted relative risk of 0.6 (95% CI: 0.4 to 1.1; p=0.103) suggests a potential reduction in negative postoperative experiences associated with preoperative anxiolysis. Very importantly, future studies are warranted to validate these observations. 

Our findings have prompted incorporating more rigorous anxiety assessments and anxiolysis in the preoperative holding bay. This aligns with the recommendations by White and Elvir-Lazo [2], who emphasize that preoperative anxiety is frequently under-assessed and suggest its routine evaluation to identify patients requiring anxiolytic interventions. Additionally, our results support prior observations that targeted benzodiazepine administration, such as midazolam, can enhance the perioperative experience without significant adverse effects [14,40,41]. Integrating midazolam into standardized perioperative protocols could improve patient comfort, minimize psychological distress, and potentially optimize recovery. Restrictive perioperative benzodiazepine strategies require active anxiety assessment and individualized perioperative support to avoid excessive perioperative distress.

Perioperative anxiety needs to be addressed in future studies. Non-pharmacological strategies, such as structured communication, perioperative coaching, music therapy, and environmental optimization, as well as pharmacological anxiolysis may be evaluated.

Limitations

This was a prospective observational, non-randomized study with no protocolized intervention, and midazolam was given at the discretion of the anesthesiology team, introducing a potential selection bias. Furthermore, the mean initial APAIS score was low, limiting the potential for a more substantial clinical improvement following the midazolam administration. Consequently, while the observed reduction was statistically significant, the absolute effect size was modest. Patients receiving midazolam were perceived as more anxious initially, potentially exaggerating its observed impact. Midazolam administration was based on the clinical judgement of the anesthesiologist, and the reasons for the clinical decision may not have been based exclusively on patients' documented preoperative anxiety levels. We believe that our prospective observational study adds important value since it reflects clinical practice in a diverse population. Additionally, the exploratory analysis was limited to a 34.9% response rate and potential recall bias due to the 9-11-month interval between surgery and follow-up. 

## Conclusions

Our data show that anxiety levels increase from the preoperative holding bay to the operating room, an effect that was mitigated in patients who received a mean (SD) of 1.9 (0.6) mg of midazolam. This anxiolytic benefit was magnified in female and younger patients. These findings underscore the importance of continuous, structured anxiety assessments.

## Figures and Tables

**Figure 6:**
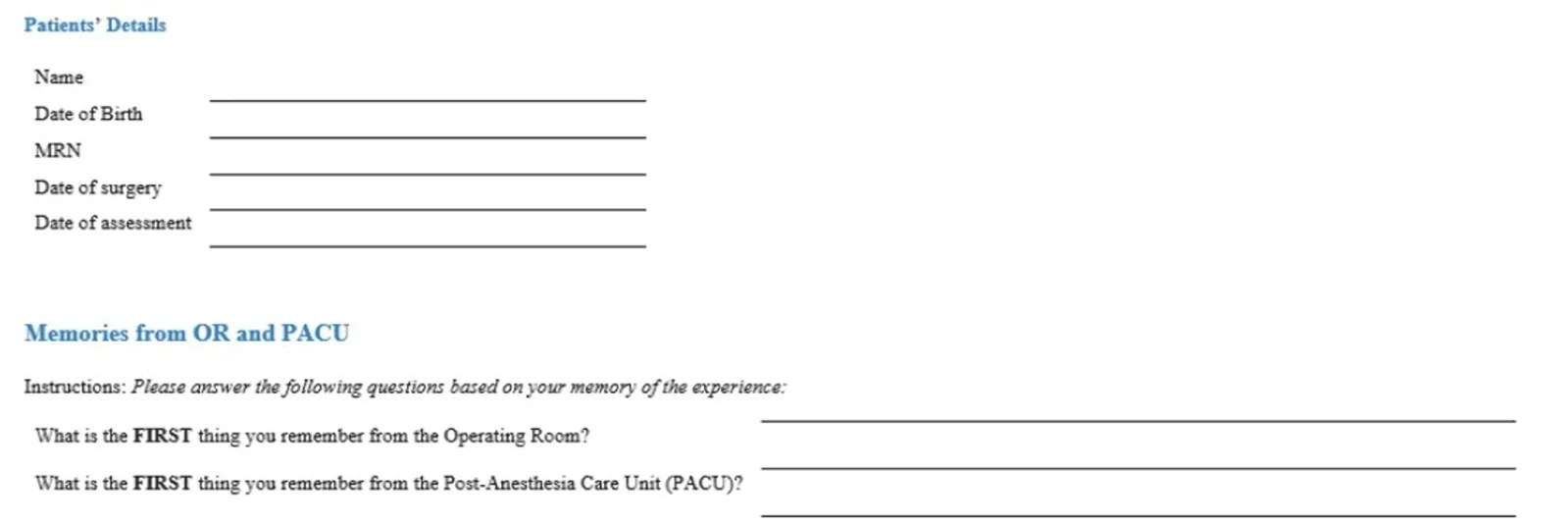
Negative stimuli questionnaire Open-ended negative stimuli questionnaire evaluating initial memories from the OR and PACU. Respondents were instructed to answer based on their recollection of the experience and to describe the first memory that came to mind for each setting. OR: operating room; PACU: post-anesthesia care unit

**Figure 7:**
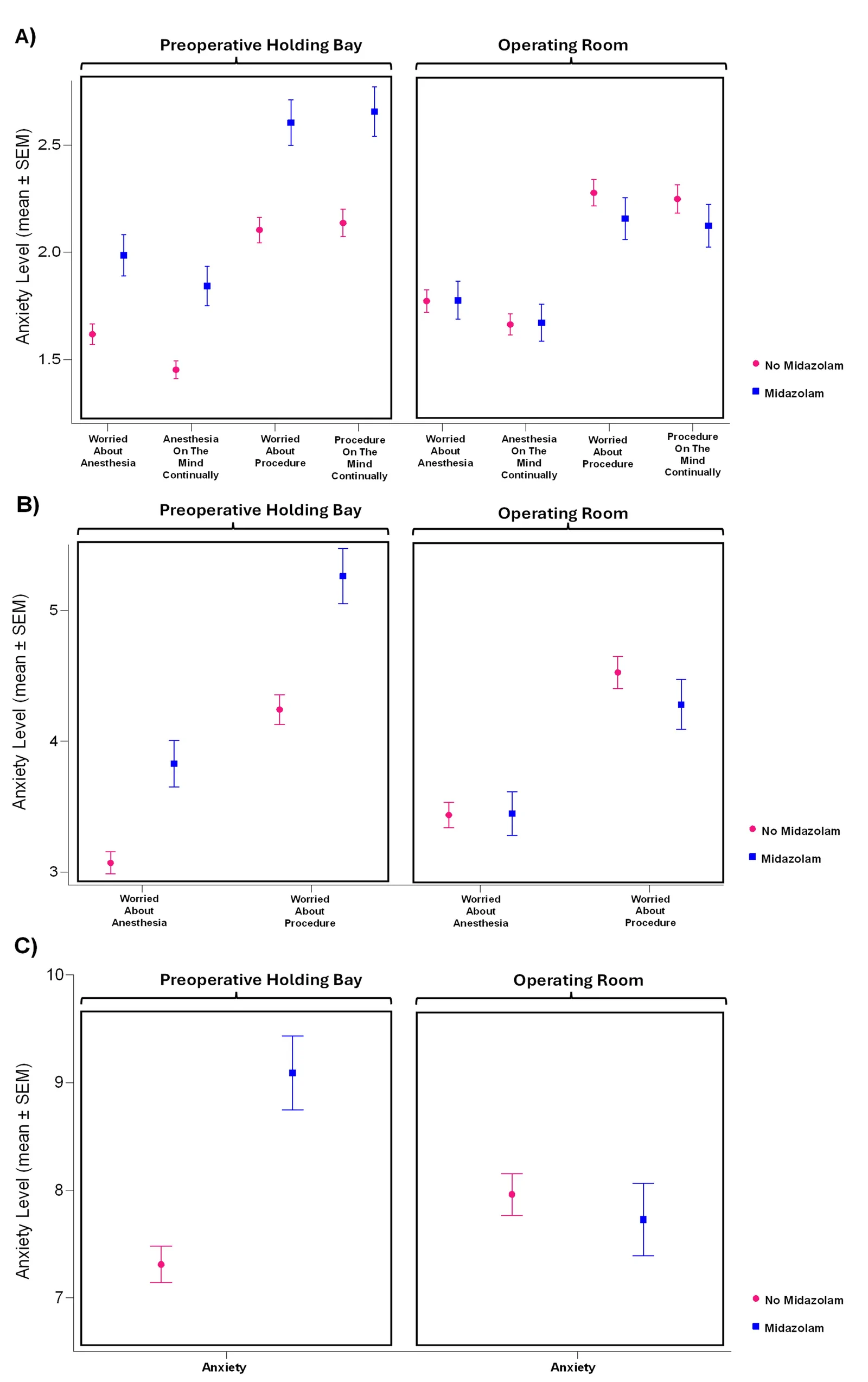
Anxiety levels in the preoperative holding bay and operating room with and without midazolam Comparisons of anxiety levels reported using the Amsterdam Preoperative Anxiety and Information Scale (mean (SEM)) in patients either administered midazolam (blue squares) or not administered midazolam (pink circles) in two perioperative settings: the preoperative holding bay and the operating room. Procedure-related anxiety was higher compared to anesthesia-related anxiety in all settings. (A) Anxiety levels across four specific concerns ("Worried About Anesthesia", "Anesthesia on the Mind Continually", "Worried About Procedure", and "Procedure on the Mind Continually") are shown for both settings. Anxiety levels increased across all domains in patients who did not receive midazolam, while they decreased in those who did. (B) Aggregated anxiety levels related to "Anesthesia" and "Procedure" are shown separately for the preoperative holding bay and the operating room. (C) Overall anxiety scores are displayed, showing that anxiety went up if midazolam was not administered but went down when it was given.

## Appendices

Appendix A

Appendix B

**Table 3:** Description and categorization of covariates Covariates are presented with their definitions and categorizations as used in the analyses.

Covariates	Definition	Categorization
Age	Age at date of surgery	Quintile 1: ≥18 and ≤39
		Quintile 2: ≥40 and ≤59
		Quintile 3: ≥60 and ≤92
Sex	Sex assigned at birth	Female/Male
Race/ethnicity	As recorded in the electronic health record	Non-Hispanic White
		Hispanic
		Non-Hispanic Black
		Non-Hispanic Asian
		Other
		Not reported/Unavailable
Charlson Comorbidity Index score	Combination of existing comorbidities	Quintile 1: =0
		Quintile 2: ≥1 and ≤2
		Quintile 3: =3
		Quintile 4: ≥4 and ≤7
		Quintile 5: ≥8 and ≤18
Cancer	History of comorbidity within one year prior to surgery	Yes/No
Congestive heart failure		Yes/No
Chronic pulmonary disease		Yes/No
Cerebrovascular disease		Yes/No
Dementia		Yes/No
Diabetes mellitus with organ failure		Yes/No
Hemiparesis		Yes/No
Diabetes mellitus without organ failure		Yes/No
Human immunodeficiency virus		Yes/No
Myocardial infarction		Yes/No
Mild liver disease		Yes/No
Mild severe liver disease		Yes/No
Peripheral vascular disease		Yes/No
Renal failure		Yes/No
Rheumatoid arthritis		Yes/No
Tumor		Yes/No
Procedural service	Hospital service performing the procedure	
Status of the procedure	Status as recorded in electronic health record	Emergent
		Urgent
		Elective
Admission status	As calculated by the difference of admission day and day of procedure	Ambulatory
		Same-day admission
		Inpatient
Anesthesia type	As recorded in the electronic health record	General
		Regional
		Monitored anesthesia care
American Society of Anesthesiologists status	As recorded in the electronic health record	≥1 and ≤4

Appendix C

**Table 4:** Description of comorbidities included in the confounder model Comorbidities used for confounder adjustment were identified using International Classification of Diseases, Ninth Revision (ICD-9) and Tenth Revision (ICD-10) diagnostic codes. Codes were obtained from electronic health records prior to the surgical procedure.

Variable	ICD-9	ICD-10
Cancer	(140.X-172.X), (174.X-195.8), (200.X-208.X), 238.6	(C00.X-C26.X), (C30.X- C34.X), (C37.X- C41.X), C43.X, (C45.X-C58.X), (C60.X- C76.X), (C81.X-C85.X), C88.X, (C90.X- C97.X)
Congestive heart failure	398.91, 402.01, 402.11, 402.91, 404.01, 404.03, 404.11, 404.13, 404.91, 404.93, 425.4, 425.5, 425.6, 425.7, 425.8, 428.X	I09.9, I11.0, I13.0, I13.2, I25.5, I42.0, I42.5, I42.6, I42.7, I42.8, I42.9, I43.X, I50.X, P29.0
Chronic pulmonary disease	416.8, 416.9, (490.X-505.X), 506.4, 508.1, 508.8	I27.8, I27.9, (J40.X-J47.X), (J60.X-J67.X), J68.4, J70.1, J70.3
Cerebrovascular disease	362.34, (430.X-438.X)	G45.X, G46.X, H34.0, (I60.X-I69.X)
Dementia	(290.X-291.X)	(F00.X-F03.X), F051.X, G30.X, G311.X
Diabetes mellitus	(250.4-250.7)	(E10.2X-E10.5X), E10.7, (E11.2X-E11.5X), E11.7, (E12.2X-E12.5X), E12.7, (E13.2X-E13.5X), E13.7, (E14.2X-E14.5X), E14.7
Hemiparesis	344.9, (341.X-344.7)	G04.1X, G11.4X, (G80.1X-G80.2X), (G81.X-G83.4X), G83.9X
Human immunodeficiency virus	(042-045)	(B20.X-B22.X), B24.X
Myocardial infarction	410.X, 412.X	I21.X, I22.X, I25.2
Liver disease	(456.0X-456.2X), (572.2-572.8)	I85.0, I85.9, I86.4, I98.2, K70.0X-K70.3X] K70.4, K70.9X, K71.1X, K 71.3X-71.5X, K71.5X, K71.7X, K72.1, K72.9, (K73.X-K74.X), (K76.X-K76.7X), I85.0, I85.9, I86.4, I98.2, K70.4, K71.1, K72.1, K72.9, (K76.5X-K76.7X), Z94.4
Peripheral vascular disease	093.0, 47.1, 437.3, (440-442), (443.1-443.9), 557.1, 557.9,	(I70.X-I71.X), I73.1X, I73.8X, I73.9X, I77.1X, I79.OX, I79.2X, K55.1X, (K55.8X-K55.9X), (Z95.8X-Z95.9X)
Renal failure	403.01, 403.11, 403.91, 404.02, 404.03, 404.12, 404.13, 404.92, 404.93, 582.X, 583.X, 585.X, 586.X, 588.0, V42.0, V45.1, V56.X	I12.0, I13.1, (N03.2X-N03.7X), (N05.2X-N05.7X), N18.X, N19.X, N25.0, (Z49.0X- Z49.2X), Z94.0, Z99.2
Rheumatologic disease	446.5, (710.X-710.4X), (725.X-726.X), 714.8, (714.X-714.2X)	(M05.X-M06.X), M31.5X, (M32.X-M34.X), M35.1X, M35.3X, M36.X

Appendix D

Appendix E

**Table 5:** Association between midazolam administration and the change in anxiety levels from the preoperative holding bay to the OR OR: operating room; AMD: adjusted mean difference

	Anxiety in the preoperative holding bay (mean (SD))	Anxiety in the OR (mean (SD))	AMD/95% CI/p-value	AMD/95% CI/p-value
No midazolam (n=555)	7.3 (4.0)	8.0 (4.6)	0.6/0.4 to 0.9/<0.001	-2.0/-2.5 to -1.5/<0.001
Midazolam (n=210)	9.1 (5.0)	7.7 (4.9)	-1.4/-1.9 to -0.9/<0.001	
